# Mpox-HIV co-infection in the mpox treatment centre in Kokolo Health District, Kinshasa, Democratic Republic of the Congo, November 2024 to March 2025

**DOI:** 10.11604/pamj.supp.2025.50.1.47275

**Published:** 2025-05-07

**Authors:** Levis Amisi Kengea, Masamba Bikoki Winnie, Ndumbi Temuangudi Vally, Jean Claude Nsinga Bungiena, Jean Jacques Kape Kalume, Anthony Mbuyi Mutombe, Blanche Ihekambangu Ngwakaha

**Affiliations:** 1 Kokolo Health District, Military Health Corps, Armed Forces, Kinshasa, Democratic Republic of Congo,; 2 School of Public Health, the University of Kinshasa, Kinshasa, Democratic Republic of the Congo,; 3 Central Military Hospital, Military Health Corps, Armed Forces, Kinshasa, Democratic Republic of Congo,; 4 Type A Operational Medical Center of the 113 Ndolo Air Base, Military Health Corps, Armed Forces, Kinshasa, Democratic Republic of Congo,; 5 Command of the Military Health Corps, Armed Forces, Kinshasa, Democratic Republic of Congo

**Keywords:** Mpox, HIV, sexually transmitted infection, Holistic care, Kinshasa, Democratic Republic of Congo

## Abstract

**Introduction:**

mpox infection is an emerging zoonosis with a significant impact on public health. HIV is an infection that often causes a decline in the immunity of patients. In the event of mpox-HIV co-infection, patients see their immunity drop further. In addition to the classic clinical manifestations, patients with mpox-HIV co-infection can develop severe forms of the disease. The objective of this study is to determine the prevalence of cases of mpox-HIV co-infection among people living with mpox in the mpox Treatment Centre (MpoxTC) in Kokolo Health District.

**Methods:**

this is a descriptive cross-sectional study conducted in the MpoxTC of the Kokolo Health District, from November 26, 2024, to March 11, 2025. Exhaustive sampling was used with 118 mpox-confirmed patients. Excel 2019 was used for the data entry, and Epi Info 7.2.6.0 was used for statistical analyses, including Chi-square and Student t tests to explore associations between variables.

**Results:**

the study revealed a prevalence of 9.32% mpox-HIV coinfection 11/118, with all infections occurring through sexual contact. In total, mpox-VIH co-infected patients were distributed in 7 health areas (HAs), of which 71.43% (5/7) were urban HAs and 28.57% (2/7) were peri-urban HAs. A prolonged duration of hospitalization (>10 days) concerned 55.09% or 65/118 mpox cases, of which 8/11 cases of mpox-HIV co-infection of coinfected patients, and 94.90% (112/118) of mpox patients had skin lesions, of which 90.90% (10/11) were cases of mpox-HIV co-infection. There has also been a gradual increase in cases of severe and critical forms of mpox-HIV co-infection over time following late diagnosis.

**Conclusion:**

these results highlight the need for systematic HIV screening in all cases of mpox to exclude any case of mpox-HIV coinfection for holistic management of both diseases. Mpox can now be considered as one of the opportunistic infections of HIV. Preventive measures, including awareness and education of mpox patients, especially on mpox-HIV coinfection, are essential to reduce the number of mpox deaths in the MpoxTC of the Kokolo Health District.

## Introduction

Mpox, formerly known as monkeypox, is an emerging viral zoonosis first reported in humans in 1970 in the Democratic Republic of Congo (DRC) [1,2]. In 2023, 14,626 cases of mpox and 654 deaths (case fatality rate of 4.5%) were reported in the DRC. These are the highest numbers ever recorded in the country and the highest figures within the WHO African Region. Of these cases, 1,461 (10%) were laboratory tested, of which 966 were positive (positivity rate of 68%) [3]. As of 26 May 2024, a total of 7,851 mpox cases have been reported in the DRC, including 384 deaths (case fatality rate of 4.9%) [1]. These cases were reported in 177 of 519 health zones (34%), in 22 of 26 provinces in the DRC (85%) [4].

HIV/AIDS is one of the stigmatizing pathologies that weaken the immunity of patients, like COVID-19 [5]. This often observed decrease in immunity, often underdiagnosed, can exacerbate the suffering of patients, lead to severe forms of the disease, reduce their quality of life, and hinder their clinical recovery, especially if treatment is delayed [6]. In this context, it becomes necessary to verify what exactly the situation may be among mpox-HIV coinfected people for better monitoring of patients, especially in regions where the disease is in its epidemic form, such as in Kinshasa.

Patients with mpox must not only cope with the symptoms of mpox but also with the problem of HIV, hence the painful skin lesions and fever, anorexia, more pronounced physical asthenia, anemia, and the social stigma associated with a disease perceived as contagious and shameful [7,8]. Mpox can worsen HIV and vice versa, with feelings of isolation mainly due to fear of stigma, creating a vicious cycle that compromises their overall well-being [9]. Despite the importance of these issues, few studies have addressed mpox-HIV coinfection.

Epidemics place considerable pressure on health systems and populations, especially if patients initially had other, especially chronic, pathologies [8,9]. In the DRC, a country facing recurrent health challenges, HIV remains a public health problem, affecting more females than males [10]. Understanding the prevalence and risk factors associated with the occurrence of mpox among PLHIV during an mpox epidemic is essential to develop effective public health interventions and inform health policies in similar contexts [11,12]. Health professionals frequently report warning signs of the need to check HIV status in hospitalized mpox patients [13,14]. However, few systematic studies have been conducted to quantify the prevalence of mpox-HIV coinfection cases in this population or to identify the factors associated with it. A better understanding of these aspects could guide the development of holistic management, adapted and integrated with existing medical care for mpox cases. Thus, this study aims to fill a major gap in the literature by exploring different mpox cases in the Kokolo MpoxTC, in Kinshasa, to determine the existence of mpox-HIV coinfection, the prevalence of mpox-HIV infection and propose measures of gestion and control, the reason for this study.

Mpox is a viral zoonosis caused by the mpox virus, belonging to the genus orthopoxvirus. Initially identified in humans in 1970, the disease was reported mainly in endemic regions of Central and West Africa. However, since 2022, unusual outbreaks have been observed in non-endemic regions, attracting global attention [2]. Clinically, mpox presents with fever, pustular skin lesions, lymphadenopathy, and systemic symptoms. The duration of symptoms varies from two to four weeks, and mortality remains low in mild forms, although it can reach 10% in some more virulent strains [15]. HIV prevalence is 1% in the DRC; it is not known in Kinshasa, let alone in the Kokolo Health District [16]. These figures highlight the importance of contextual factors in the occurrence of mpox in PLHIV.

Emerging and infectious diseases, particularly those associated with visible symptoms and high stigma, such as mpox, are frequently linked to severe disease in patients with chronic conditions. Studies conducted during previous pandemics and epidemics, such as HIV/AIDS, Ebola, and more recently COVID-19, have reported a prevalence of severe disease with an increase in anxiety and depressive disorders, as well as HIV, among affected populations [17,18]. Underlying mechanisms include fear of illness, social isolation, stigma, and uncertainty about clinical outcomes. These disorders, when left untreated, can exacerbate patient suffering and slow recovery [6,19]. Although data specific to the DRC is limited, similar trends are likely observed there, given existing vulnerabilities.

Patients with chronic or infectious diseases during epidemics are particularly vulnerable. A study conducted in Kinshasa revealed that 35% of drug-resistant tuberculosis patients had poor quality of life, associated with age, type of tuberculosis, delay in psychosocial support, tuberculosis-HIV coinfection, severe form of the disease, perceived stress, and anxiety-depression [20]. Mpox-HIV coinfection is possible where the mpox epidemic affects all strata, although males and adults were more affected [21]. Furthermore, it is relevant to broaden the scope of action to HIV status verification interventions in mpox patients. However, very few studies have explored its implications on mpox-HIV coinfection [21]. With the increased attention paid to mpox, few studies have explored its implications for HIV. Associated factors included the severity of skin lesions, prolonged duration of isolation, and perceived stigmatization by patients [21,22].

The combination of socioeconomic factors, poverty, armed conflict, and health crises creates a fertile ground for HIV contamination by mpox patients and vice versa [15,17-19]. Training healthcare professionals in the detection and management of mpox and HIV, as well as community awareness, are essential steps to mitigate the psychological impact of epidemics [15,17-19].

A thorough exploration of these aspects in the Kinshasa context was essential to inform health policies and guide targeted interventions. By integrating mpox-HIV surveillance methods, this study aims to fill a critical gap in the literature and offer recommendations for the holistic management of patients with mpox-HIV coinfection. The overall objective of this work is to investigate cases of mpox-HIV coinfection in the Kokolo mpox treatment center from November 2024 to March 2025. The specific objectives were to confirm the existence of cases of mpox-HIV coinfection, determine the prevalence of mpox-HIV coinfection, describe the sociodemographic and clinical characteristics of patients, and propose management and control measures.

## Methods

**Study design:** we conducted an exhaustive cross-sectional descriptive survey to determine the prevalence of mpox-HIV coinfection cases and to propose management and control measures.

**Setting:** this study was conducted at the Kokolo Health District Mpox Treatment Center (MpoxTC), which is an mpox treatment center located in a military camp and a military health zone to take care of all the detainees in the dungeons and prisons of the city of Kinshasa, soldiers suffering from mpox, their dependents but also the surrounding population in Kinshasa, Democratic Republic of Congo.

**Participants:** participants consisted of patients with confirmed mpox treated at the Kokolo Health District mpox Treatment Center between 26th November 2024 to 11th March 2025.

**Inclusion criteria:** patients aged 18 years and older; patients with a confirmed diagnosis of mpox, and Patients who provided informed consent.

**Exclusion criteria:** patients with cognitive impairment making questionnaire administration impossible; Patients not hospitalized at the Kokolo Health District mpox Treatment Center, or refusal to participate.

**Sampling and study size:** we exhaustively sample all patients admitted at the Kokolo Health District mpox Treatment Centre during the study period, using the hospitalization registry. Thus, all 118 patients were included in the study.

**Data sources/measures:** data related to the variables of interest were collected using a pre-established structured electronic questionnaire. Data were collected during consultations or hospitalizations at the Kokolo Health District mpox Treatment Center and from the patient registry.

**Definition of concepts:** mpox case: any patient presenting with typical skin lesions with a mpox diagnosis confirmed by a PCR test.

*HIV case:* any case detected by RDT according to the planned algorithm and then confirmed by ELISA. The algorithm is such that the three tests to be used first are determined, Stat-Pak, and Unigold. For certainty, we use Innolia (ELISA).

*Mild case mpox:* any confirmed case of mpox with fewer than 25 rashes on the body without any criteria of severity.

*Moderate case mpox:* any confirmed case of mpox with 25 to 99 rashes on the body without any criteria of severity.

*Severe case mpox:* any confirmed case of mpox with 100 to 250 rashes on the body with of the following severity criteria child under 5 years of age, pregnant woman, immunocompromised case (HIV, TB, cancer, severe malnutrition, etc.), case with involvement of major organs (heart, brain, lungs, etc).

*Critical case mpox:* any confirmed case of mpox with 250 rashes on the body with of the following severity criteria child under 5 years of age, pregnant woman, immunocompromised case (HIV, TB, cancer, severe malnutrition, etc.), case with involvement of major organs (heart, brain, lungs, etc).

**Study variables:** sociodemographic characteristics, clinical data, positive HIV diagnosis among mpox patients in the MpoxTC of the Kokolo Health District. Data were collected on the electronic data collection platform (KoboToolbox) and extracted from the server. After cleaning, consistency, and completeness checks, they were then saved in an Excel 2019 spreadsheet, then exported to the EpiInfo 7.2.6.0 data analysis software.

**Statistical methods:** we analyse the distribution of clinical and socio-demographic parameters among patients using descriptive statistics summarized as (mean, median, standard deviation, and range) for continuous variables, and as frequency and proportion tables for categorical variables. The 95% confidence level was used, with an α threshold of significance less than 0.05 considered significant.

**Possible bias:** possible reporting bias, as we cannot determine whether cases of mpox-HIV coinfection were already HIV cases before the RDTs and ELISA tests during this study. It is therefore possible that patients omitted or modified certain information due to shame. To minimize selection bias, we used exhaustive sampling. For confirmation bias, we only considered positive results after ELISA. Correcting or minimizing these biases requires rigorous study planning, representative samples, systematic data collection, and thorough analysis.

**Ethical considerations:** approval to conduct the research was obtained from the French National Health Ethics Committee (No. 630/CNES/BN/PMMF/2025 of 02/16/2025). The basic principles of health research ethics were respected during data collection and the presentation of results. Mission orders were obtained from the Kokolo Health District before the visit to the Kokolo Health District MpoxTC. Consent was obtained from all patients at the outset, and confidentiality and anonymity were guaranteed during data collection and presentation of results.

## Results

**Number of HIV cases detected at the Kokolo MpoxTC:** the prevalence of mpox-HIV coinfection in the Kokolo Health District’s MpoxTC is 9.32%, or 11/118 mpox cases. The majority of mpox cases, 66.10% (78/118), were recorded in peri-urban health areas (Has), in contrast to the large proportion of Mpox-HIV coinfection cases found in urban health areas (Table 1).

**Table 1:** number of HIV cases detected among patients with mpox in the Kokolo Health Zone, MpoxTC, November 2024 to March 2025

Variables	Number of mpox cases	Diagnosed HIV cases among mpox cases
**MpoxTC**		
**Kokolo**	118 (100%)	11 (9.32%)
**Health area**		
**Peri-urban**	78 (66.10%)	02 (2.56%)
**Urban**	40 (33.90%)	05 (12.5%)
**Total**	118 (100%)	11 (9.32%)

**Clinical characteristics:** the average length of stay at the Kokolo Health District MpoxTC was 10.7 ± 5.4 days when 49.2%, or 58/118, constituting the majority of mpox cases, had spent 7 to 14 days of hospitalization at the MpoxTC. The majority of patients with mpox-HIV coinfection, or 72.72% (8/11 comorbid mpox-HIV patients), had a stay of more than 14 days of hospitalization in the Kokolo Health District MpoxTC. Skin lesions were found in 112 mpox cases (94.9%) and fever in 106 mpox cases (89.8%) (Table 2).

**Table 2:** distribution of cases by sociodemographic characteristics of HIV cases among patients with mpox and clinical characteristics in the MpoxTC of the Kokolo Health District

Variables	Total mpox cases; N=118 (%)	HIV		
		Positive; N=11 (%)		Negative; N=107 (%)
**Sex**				
Female	48 (40.68)	07 (14.58)		41 (85.42)
Male	70 (59.32)	04 (5.71)		66 (94.29)
**Age group (years)**				
<25	25 (21.19)	04 (16.00)		21 (84.00)
25-34	64 (54.24)	04 (6.25)		60 (93.75)
35-44	21 (17.8)	02 (9.52)		19 (90.48)
>44	08 (6.78)	01 (12.50)		07 (87.50)
**Education level**				
No education	37 (31.36)	05 (13.51)		32 (86.49)
Primary	65 (55.08)	04 (6.15)		61 (93.85)
Secondary	12 (10.17)	02 (16.67)		10 (83.33)
Higher education/university	04 (3.39)	00 (00.00)		04 (100.00)
**Marital status**				
Single	75 (63.56)	08 (10.67)		67 (89.33)
Partnered	43 (36.44)	03 (6.98)		40 (93.02)
**Occupation**				
Military dependents	08 (6.78)	01 (12.50)		07 (87.50)
Military	99 (83.90)	08 (8.08)		91 (91.92)
Civilians outside	11 (9.32)	02 (18.18)		09 (81.82)
**Length of stay at CTMpox**	Average: 10.7± 5.4 days			
<7 days	45 (38.14)		01 (2.22)	44 (97.78)
7-14 days	58 (49.15)		04 (6.90)	54 (93.10)
>14 days	15 (12.71)		06 (40.00)	09 (60.00)
**Clinical symptoms**				
Fever	106 (89.83)		09 (8.49)	97 (91.51)
Chills	58 (49.15)		06 (10.34)	52 (89.66)
Headache	82 (69.49)		07 (8.54)	75 (91.46)
Asthenia	76 (64.41)		08 (10.53)	68 (89.47)
Lymphadenopathy	102 (86.44)		08 (7.84)	94 (92.16)
Arthralgia/myalgia	67 (56.78)		05 (7.46)	62 (92.54)
Skin lesions	112 (94.92)		11 (9.82)	101 (90.18)
Sore throat	97 (82.20)		09 (9.28)	88 (90.72)
Rectal symptoms	21 (17.80)		04 (19.05)	17 (80.95)

**Distribution of HIV cases by sociodemographic characteristics:** male mpox cases are the most affected at 59.32% or 70/118, while cases of mpox-HIV coinfection and deaths were mainly among female subjects, respectively with 14.58% (7/48) and 4.17% (2/48). The age group most affected by mpox is that of 25 to 34 years, with 54.24% (64/118), and cases of mpox-HIV coinfection are predominantly among subjects aged 18 to 24 (16% or 4/25) and over 44 years (12.50% or 1/8); the latter had even died. The majority of mpox cases had a primary education level (55.08%, either 65/118), while the majority of mpox-HIV coinfection cases had no education level (13.51%, either 5/37), and deaths were more recorded among subjects with a primary education level (3.08%, either 2/65). The majority of mpox cases, mpox-HIV coinfection cases, and deaths were among those living alone, with 63.56% either 75/118; 10.67% either 8/75; and 2.67% either 2/75, respectively. Regarding occupation, military personnel were the most affected among mpox cases (83.90%, or 99/118), but mpox-HIV coinfection cases and deaths were more common among military dependents, with 12.50% or 1/8 (Table 2).

**Evolution of mpox cases in the MpoxTC of the Kokolo Health District:** the epidemic is still in its ascending phase with occasional ups and downs.

**distribution of patients by disease level in the MpoxTC:** among the cases of mpox-HIV coinfection, the majority experienced the third level mpox case with 72.73% or 8/11, while the majority of cases with the mild form (first level) were found among the non-HIV mpox cases (Table 3).

**Table 3:** distribution of patients by level of disease or gravity in the Kokolo MpoxTC

Disease severity level	HIV-mpox cases; N (%)	Non-HIV mpox cases; N (%)	Total; N (%)
Mild case mpox	01 (9.09%)	54 (50.47%)	55 (46.61%)
Moderate case of mpox	02 (18.18%)	38 (35.51%)	40 (33.90%)
Severe or critical case of mpox	08 (72.73%)	15 (14.02%)	23 (19.49%)
**Total**	11 (9.32%)	107 (90.68%)	118 (100%)

## Discussion

**Number of cases of mpox-HIV coinfection at the Kokolo MpoxTC:** our result shows a high hospital frequency of HIV cases among patients with mpox in the Kokolo MpoxTC (9.32%). The prevalence of HIV in Kinshasa is 1.2% and in the DRC 1%, while that of the Kokolo Health Zone is not yet known [16]. This is explained by the fact that HIV constitutes a breeding ground for other infectious diseases and vice versa [8,9]. The urban health areas (Kokolo1, Kokolo2, Logistics Base, Tshatshi, and Ndolo) recorded a higher proportion of cases of mpox-HIV comorbidity (71.43%, or 5/7) compared to the peri-urban health areas (28.57%, or 2/7). This difference could be attributed to the fact that there is only a small concentration of soldiers and their dependents in the military camps on the outskirts of the city.

**Clinical characteristics of patients:** our results show that the most frequently reported clinical symptoms among mpox cases include skin lesions (94.9%), fever (89.8%), and lymphadenopathy (86.4%). This corroborates data from several authors [23-25]. The majority of patients were hospitalized for between 7 and 14 days (49.2%, or 58/118), and 72.72%, or 8/11 comorbid mpox-HIV patients, experienced a longer hospital stay in the Kokolo MpoxTC, which could indicate a link between the length of hospitalization and the form of the disease [26-28].

**Sociodemographic characteristics of mpox-HIV coinfection cases:** women are slightly more affected by mpox-HIV coinfection than men. This may be explained by the fact that HIV generally tends to affect women much more. The age groups <25 and 25-34 years accounted for the majority of mpox-HIV cases, with 36.36%, or 4/11 each, which could be related to greater sexual activity associated with age. Furthermore, military personnel are the most affected (72.72%, or 8/11). This finding highlights the role of socioeconomic factors in mpox-HIV coinfection among patients, and occupations can increase risk. The majority of mpox cases had a primary education level and lived alone, while the majority of mpox-HIV coinfection cases had no education level, and deaths were more recorded in subjects with a primary education level and aged over 44 years.

**Prevalence of coinfection mpox-HIV cases:** our results reveal that 9.32% of patients hospitalized for mpox also have HIV, which shows that mpox may be one of the opportunistic infections of HIV. Severe and critical mpox cases were mostly found among cases of mpox-HIV coinfection, with 3 deaths recorded. HIV was discovered incidentally in the majority (6/11), especially following the severe and critical mpox case table, in these different mpox cases who were unaware of their HIV serological status. This corroborates with Fischer et al. (2022) who stated that HIV is often a pathology that paves the way for other epidemics and vice versa, and accelerates the occurrence of deaths in cases of coinfection [8,29].

**Limitations:** the study was conducted in the only mpox treatment center in the Kokolo Health Zone at a time when there are currently five mpox treatment centers in the city of Kinshasa. This may be a limitation for the generalization of these results, but it should be noted that this is the first study carried out in this direction in the city of Kinshasa and includes all patients in the treatment center. Furthermore, the perceived stigmatization of suspected cases compromised contact tracing efforts, as those affected were often reluctant to engage with investigation teams and provide accurate information.

**Conclusion**

The objective of this study was to investigate mpox cases by determining the existence of mpox-HIV coinfection cases, determining the prevalence of mpox-HIV coinfection cases in the MpoxTC in the Kokolo health district, and proposing measures of prevention and control. The results show a high prevalence of mpox-HIV coinfection cases. These are patients with mpox-HIV coinfection with severe disease leading to death. Furthermore, all these results demonstrate the importance of verifying the strong association between mpox infection and HIV, particularly influenced by clinical (severity of symptoms, length of hospitalization) and socioeconomic (unemployment, poverty, and stigma) factors. These results highlight the need to check the HIV profile of patients admitted to the mpox treatment centre in all mpox cases before starting treatment to prevent worsening of the disease. Du fait que le VIH se transmet par la voie sexuelle, même constat pour le sous-clade Ib du mpox, avec cette prévalence de 9.32%, il y a lieu de mener des études plus poussées avec un échantillon plus grand pour savoir si le mpox, surtout son sous-clade Ib, n´est pas une des infections opportunistes du VIH.

### What is known about this topic

- Several provinces in the Democratic Republic of Congo and many African countries have reported cases of Mpox and HIV;
- Mpox and HIV can easily coexist in a patient.

### What this study adds

- This study shows that there is a significant prevalence of cases of mpox-HIV co-infection in CTMpox Kokolo and the interest in checking for HIV in any mpox case.
